# Hydrogels as artificial matrices for cell seeding in microfluidic devices

**DOI:** 10.1039/d0ra08566a

**Published:** 2020-12-08

**Authors:** Fahima Akther, Peter Little, Zhiyong Li, Nam-Trung Nguyen, Hang T. Ta

**Affiliations:** Australian Institute for Bioengineering and Nanotechnology, The University of Queensland Brisbane Queensland Australia hangthuta@gmail.com; Queensland Micro- and Nanotechnology Centre, Griffith University Brisbane Queensland Australia h.ta@griffith.edu.au; School of Environment and Science, Griffith University Brisbane Queensland Australia; School of Pharmacy, The University of Queensland Brisbane Queensland Australia; School of Mechanical Medical & Process Engineering, Queensland University of Technology Brisbane Australia

## Abstract

Hydrogel-based artificial scaffolds play a vital role in shifting *in vitro* models from two-dimensional (2D) cell culture to three-dimensional (3D) cell culture. Microfluidic 3D cell culture systems with a hydrogel matrix encourage biomedical researchers to replace *in vivo* models with 3D *in vitro* models with a cellular microenvironment that resembles physiological conditions with greater fidelity. Hydrogels can be designed as an artificial extracellular matrix scaffold for providing spatial orientation and promoting cellular interactions with surroundings. Selecting the appropriate hydrogels and their fabrication techniques are the key to mimic the *in vivo* mechanical environment. Moreover, combining a microfluidic technique with a hydrogel-based 3D cell culture system can create a complex and controlled microenvironment for the cells by placing small biosamples inside the microchannel. This paper provides an overview of the structural similarities of the hydrogels as an extracellular matrix (ECM), their classification and fabrication techniques as an ECM, and their use in microfluidic 3D cell culture systems. Finally, the paper presents the current challenges and future perspectives of using hydrogel scaffolds in microfluidic 3D cell culture systems.

## Abstract 2

Hydrogel-based artificial scaffolds and its incorporation with microfluidic devices play a vital role in shifting *in vitro* models from two-dimensional (2D) cell culture to *in vivo* like three-dimensional (3D) cell culture

## Graphical abstract

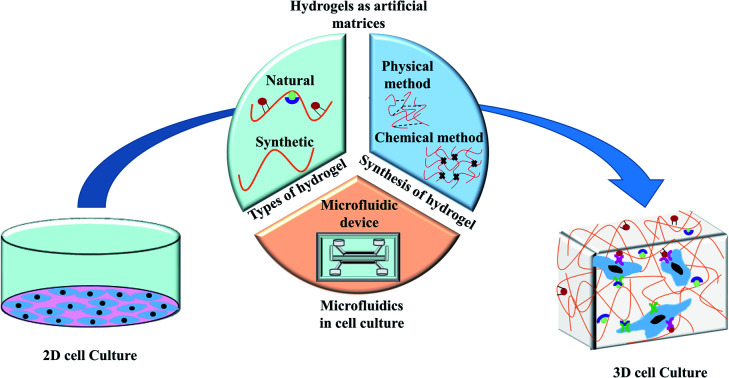

## 1. Introduction

The cellular microenvironment plays a vital role in substantial cellular morphology and the activation of a wide range of factors for regulating cell growth, proliferation, and migration. The extracellular matrix (ECM) is the non-cellular structural support of the cell, which provides the spatial orientation and tissue-specific biochemical and biophysical modulation for cellular functions such as morphogenesis and homeostasis.^1^ In conventional two-dimensional (2D) cell culture systems, cells are grown on flat Petri dishes, flasks, or tubes where only the nutrition medium provides for the cell growth. Cells mostly grow as a monolayer on the surface and these flattened cells could receive cell signals only at their ventral surface, which might alter the responses of different cellular functions.^2^ Moreover, lack of cell–cell and cell–extracellular matrix interactions can also change the cellular morphology, develop abnormal polarization, deviate phenotypic expression, and/or genotypic features.^3^

Shifting from 2D cell culture to three-dimensional (3D) cell culture lays a foundation for advancing biomedical research. In a 3D cell culture, an artificial cellular microenvironment is created as a biological scaffold to provide mechanical support for cell growth by promoting cellular interactions with the surroundings. Inventing the appropriate culture conditions might allow the researchers to have a better understating of cell biology.^3^ Human glioblastoma cells were grown in a 3D collagen scaffold to develop an *in vitro* drug screening platform.^4^ This study compared the cellular morphology and biochemical expression between the 3D scaffold cell culture and the conventional 2D culture on dishes. Cells grown in 3D scaffold showed more *in vivo* like proliferation, better cell–cell and cell–matrix interaction, greater degree of dedifferentiation and quiescence in contrast to 2D culture. In addition, glioma cells in 3D scaffold exhibited enhance chemotherapeutic resistance and expression of *O*6-methylguanine DNA methyltransferase (MGMT) compared to the cells grew in 2D culture.

To date, different biomaterials such as patterned glass substrates, elastomeric films, hydroxyapatite ceramics, hydrogels, and fibrillar foams are employed as the alternative of physical scaffold for cells. These materials create the complexity, mechanical support, composition, and structural orientation similar to the native tissue during the *in vitro* cell culture process.^5^ Among these materials, biocompatible hydrogels have gained popularity because of their cross-linkable, highly hydrated porous network for the cellular organization, promoting mechanical stiffness, and developing cytocompatible native ECM like structure.^6^

Combining a microfluidic system with a hydrogel matrix 3D cell culture is a promising approach in cellular biology, tissue engineering, and biomedical research. The approach reduces the size of bio-samples and allows for precise control over multicellular microenvironment. A microfluidic system, also known as “lab-on-a-chip”, enables the study of well controlled fluid flowing through the microchannels. It is possible to create a complex and controlled microenvironment for the cell culture inside a microchannel by regulating the shear stress and strain.^7^ On the other hand, the tunability of the porosity and elasticity of a hydrogel makes it suitable for serving as an *in vitro* matrix for organ-specific 3D cell culture systems.^8,9^ This is advantageous because the spatial distribution of signal gradients inside a microchannel can be well controlled by continuous perfusion of liquid.

This review paper discusses different types of hydrogels and their relevant characteristics for serving as the extracellular matrix. We discuss the hydrogels commonly used as cellular scaffolds in microfluidic cell culture systems, and also detail their advantages and limitation. Finally, we present the current challenges and future perspectives of hydrogel-based microfluidic 3D cell culture systems.

## 2. Hydrogels as extracellular matrix (ECM)

**Fig. 1:**
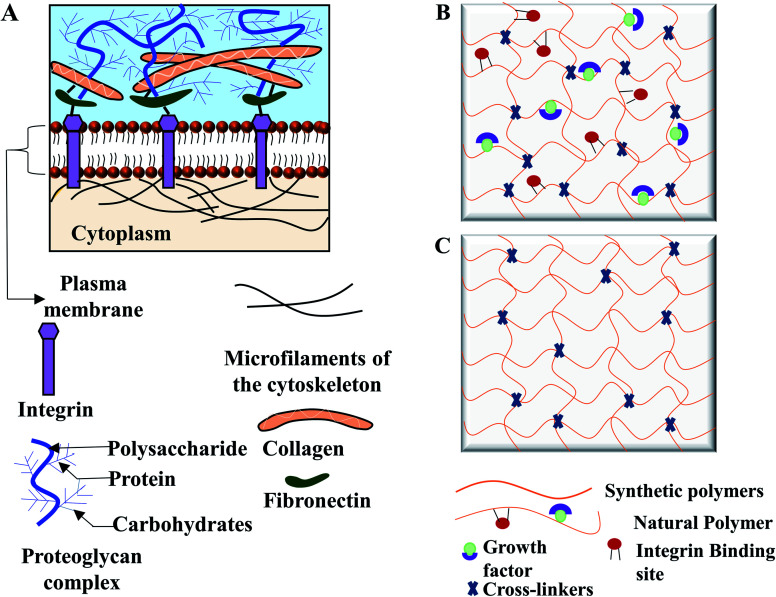
Structural similarities between extracellular matrix (ECM) and hydrogels. (A) ECM is consisted of three groups of macromolecules: structural proteins (collagens), proteoglycans, and glycoproteins (fibronectin) to form the matrix for cell attachment. Proteoglycan and structural fibrous proteins fill the cells' interstitial space to provide the mechanical and biological supports. Hydrogel scaffold from (B) natural polymers and (C) synthetic polymers. Fibrous hydrogel polymers link by interconnected microscopic pores to provide mechanical and biological cellular support.

ECM is the non-cellular self-ensemble of macromolecules, glycosaminoglycan, and fibrous protein such as collagen, fibronectin, laminin (Fig. 1A). ECM fills the extracellular space between cells to provide structural support to mammalian cells. ECM serves as a regulatory modulator for critical cellular functions, contributing to morphogenesis and regeneration of tissues.^10–12^ ECM also facilitates gas and nutrition exchange between the cell and its environment, removing metabolic waste, and regulating signal transduction pathway.^6,13^

Hydrogels are considered as the 3D analogy to native ECM due to their swelling characteristics, the high-water content and the low elasticity. Hydrogels are hydrophilic 3D cross-linked polymeric networks, consisting of interconnected microscopic pores, which can absorb biological fluid up to 99% of its volume.^14^ The amount of the absorbed fluid in the swollen hydrogel depends on the nature of the polymers and the developed polymeric network. Furthermore, the interfacial tension of hydrogels with water and biological fluids simulates the nature of the most soft tissues.^13,15,16^ Hydrogel as an artificial matrix should provide appropriate mechanical, chemical, and biological support for cell growth and maintenance. To have the similar structure of mammalian ECM, hydrogels must possess a hydrated protein and polysaccharide network.^15,17^ Like native ECM, hydrogels from natural polymers contain growth factors and integrin binding sites (Fig. 1B) for promoting cellular functions. In contrast, hydrogels from synthetic polymers lack of integrin binding ligands (Fig. 1C) but can maintain cell viability.^15^ A wide variety of hydrogels could be prepared in mild and biocompatible conditions, with possible modification for desired cell adhesion, viscoelastic moiety, and degradability.^5^ Hydrogels can be designed and modified to simulate the physicochemical properties of a target tissue. Cell adhesiveness of hydrogel can be modified by peptide and protein immobilization. Furthermore, by introducing new functional groups, matrix stiffness can be tuned for cellular support, and pore architecture can be optimized to promote tissue formation.^6^

## 3. Types of hydrogels used in microfluidic devices

**Fig. 2:**
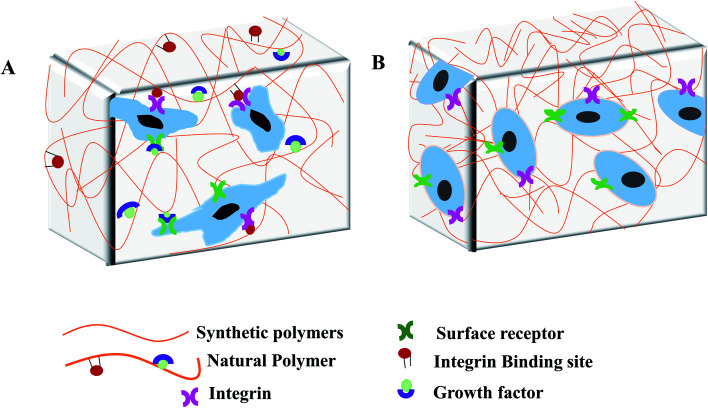
Hydrogel based artificial scaffold for cell culture. (A) Scaffold, composed of natural polymers, provides the cellular support by enabling the cells to bind with a variety of different integrin-binding sites and growth factor and regulates cell behaviour through activating signalling cascade. (B) Scaffold, composed of synthetic polymers, lacks growth factors and integrin binding sites that only provides the mechanical support for cell growth.

Hydrogel-based devices facilitate the cellular microenvironment for simultaneous loading and attachment of cells as well as nutrient supply and waste removal. These processes occur under physiologically relevant shear conditions and can be visualised by real-time microscopy.^18,19^ Hence, microporous hydrogels could generate fluid pathways throughout the 3D scaffold that accelerate the distribution of nutrients.^20^ Studies indicate the importance of hydrogels because of their capability to form complex networks and well controlled properties.^21,22^ Polymers of natural and synthetic origin are used as hydrogels in microfluidic devices.^23,24^ In general, natural hydrogels are typically formed from biologically derived precursors such as ECM proteins: collagen, fibrin, and/or hyaluronic acid, along with the polymers derived from natural sources such as chitosan and alginate.^25^ The main advantages of natural hydrogels are their biocompatibility and bioactivity. Natural hydrogels show enhance cellular attachment, proliferation and viability due to the presence of endogenous factors^26^ (Fig. 2A). However, the disadvantages of natural hydrogels are the difficulties in tuning mechanical and biochemical presentation, rapid degradability, and potential for contamination. Furthermore, batch to batch consistency is difficult to maintain in the production of natural hydrogels, that might affect the cellular behaviour and experimental reproducibility.^15^

Hydrogels derived from non-natural molecules are known as synthetic hydrogels. Commonly used synthetic hydrogels are poly(ethylene glycol), poly(vinyl alcohol), polyacrylamide, poly(aspartic acid) and poly(2-hydroxy ethyl methacrylate).^23,27^ The main advantages of synthetic hydrogels are the tunability and batch-to-batch consistency in large-scale production. These biologically inert synthetic hydrogels have limited use because of the lack of endogenous growth factors needed for cell growth (Fig. 2B). However, bioactive molecules such as protein, enzyme and growth factors can be incorporated into the synthetic hydrogels network to mediate specific cell functions.^28,29^ Synthetic hydrogels can also be chemically modified to add beneficial properties such as changing porosity and stiffness; improving stability, biocompatibility and degradability; and tuning mechanical strength for different cellular applications.^30^

## 4. Synthesis and properties of hydrogels as 3D scaffolds

**Fig. 3:**
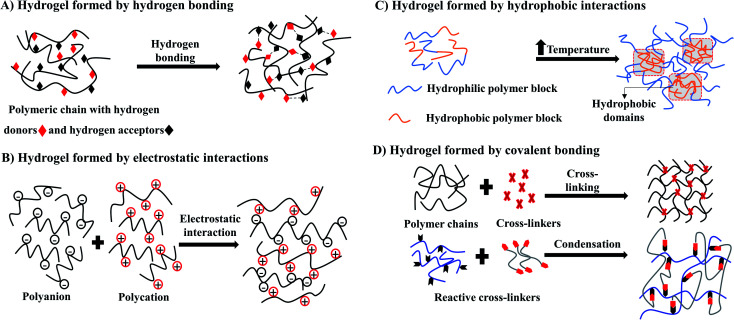
Schematic illustration of synthesis of hydrogel by physical methods (A–C) and chemical method (D).

Hydrogels can be formed by crosslinking polymeric chains through physical or chemical methods.^31^ Physical hydrogels are ionotropic, formed through molecular entanglements and secondary forces such as hydrogen bonds, crystallite formation, electrostatic interactions, and hydrophobic (Fig. 3A–C), which are often reversible. An advantage of physical hydrogels is their biocompatible nature due to the absence of chemical cross-linkers that may cause cell toxicity.^32^ However, the inflexibility towards gelation time, pore size, and chemical functionalization may lead to inconsistent performance *in vivo*.^33,34^

Conversely, chemical hydrogels formed by irreversible covalent crosslinking that is mainly induced by the high energy radiation, the addition of different crosslinking agents, free radical polymerization, and condensation polymerization (Fig. 3D) for the rapid sol–gel transition.^33^ Unlike physical hydrogels, chemical hydrogels are stable against degradation. Gelation time and pore size could be easily modified by changing chemical functionalization. However, these chemical initiators often cause toxicity to cells. Thus, when selecting synthetic hydrogels for cell culture, it is important to use non-toxic initiators to minimize cell toxicity and preserve cell function. Other considerations when using chemical hydrogels are the biocompatibility of crosslinking procedures, polymerization time, and the nature of the reagents used for the specific cell types.^33,34^

**Fig. 4:**
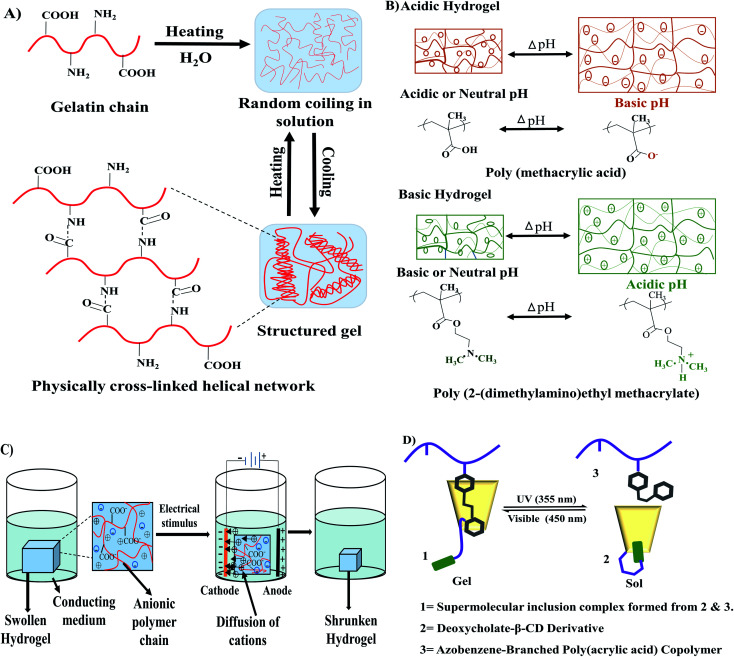
Stimuli-responsive hydrogels. (A) Thermoresponsive sol–gel transition of gelatin. (B) pH-sensitive swelling–deswelling behaviour of acidic and basic hydrogels redrew from Kocak *et al.*^42^ (D) Influence of applied electric filed on hydrogel reconstructed from Qureshi *et al.*^43^ (C) Photosensitivity in the sol–gel transition of azobenzene-based hydrogel redrew from Zhao *et al.*^41^

Stimuli-responsive hydrogels, also known as smart hydrogels, respond to external stimuli to organize their internal architectural orientation. Stimuli responsiveness can be achieved by introducing a special component in the polymeric chain that has the ability to respond to a particular signal. This component could be the specific chemical structure of the polymeric chain or could be added externally in the polymeric network.^35^ Different types of physical and biochemical stimuli such as temperature, light, magnetic field, electric field, ultrasonic wave, and pH variation are responsible for some stimuli-sensitive hydrogel fabrication. Thermoresponsive hydrogels work by changing the equilibrium between the hydrophobic and hydrophilic segments in respond to temperature changes. Thermoresponsive hydrogels can be formed by low critical temperature (LCST) with hydrogel formation undergoing phase separation and upper critical solution temperature (UCST) with hydrogels formed by heating.^36^ A small temperature change can initiate hydrophobic or hydrophilic interactions between hydrophobic or hydrophilic polymer segments respectively for inducing the sol–gel transition (Fig. 4A).^30^ Examples of thermoresponsive hydrogels are gelatin, collagen, cellulose, chitosan, starch, carrageenan, hyaluronic acid, xanthan, xyloglucan, elastin, and dextran.^37^ Some of the thermoresponsive hydrogels are reversible, especially the naturally occurring hydrogels such as gelatin, carrageenan, agarose.

pH-responsive hydrogels showed their characteristic features at a specific pH. The swelling or contraction of pH-sensitive hydrogels occurs in response to the change of the pH value in the system (Fig. 4B). All pH-responsive polymers must contain pendant acidic or basic groups which either accept or donate protons. Swelling of the anionic hydrogels occurs at the basic medium because the ionization of the pendant acidic groups takes place at a high pH value. However, the cationic hydrogels swell at low pH value because the protonation of amino/imine group occurs in an acidic medium.^38^ Natural hydrogels such as chitosan, guar gum, carrageenan, dextran, xanthan, cellulose, alginate, and synthetic hydrogels such as poly(acrylic acid), polyacrylamide, polyvinyl alcohol, polyethylene glycol could be used as the base materials for pH-sensitive hydrogels.^37^

Electro-sensitive hydrogels change their internal polymeric bonding by swelling/shrinking when exposed to an applied electric field (Fig. 4C). Most of electro-responsive hydrogels are polyelectrolytes that contain ionizable groups in their backbone or in the polymeric side chains. Synthetic polymers, used in the preparation of electro-responsive hydrogels, are polyvinyl alcohol, polypyrrole, polyaniline, polythiophene, acrylic acid/vinyl sulfonic acid, and sulfonated polystyrene.^39^ In contrast, natural polymers such as alginate, hyaluronic acid, and chitosan can also be blended with synthetic polymers to prepare such hydrogels.^35,40^

Light sensitive hydrogels are promising materials due to their easy activation by a particular light wavelength where the source can be remote and non-invasive. Zhao *et al.*^41^ showed that a hydrogel prepared of a deoxycholic acid-modified β-cyclodextrin derivative and an azobenzene-branched poly(acrylic acid) copolymer could efficiently convert at 355 nm wavelength from gel to sol while it was completely recovered from sol to gel at 450 nm irradiation (Fig. 4D).

Cell attachment is regulated by the tissue-specific ECM mechanical properties such as stiffness, stress, and strain.^44^ Elastic modulus of different types of tissue ranges from less than 1 kPa to 4 GPa, *e.g.* >1 kPa (brain, lung, breast), 1–10 kPa (endothelial tissue, muscle), 100 kPa (pre-calcified bone), 1 MPa (cartilage), and 2–4 GPa (bone).^45,46^ In *in vitro* hydrogel-based 3D cell culture, substrate mechanical properties could influence cell adhesion, spreading, migration, and cytoskeleton assembly.^47^ The scaffold architecture of the hydrogels is not only important for the cellular adhesion but also for the diffusion of nutrients, signalling molecules, and other required moieties presented into the culture environment.^48^ The mechanical properties of hydrogels, including elasticity, swelling, pore size, and surface roughness, can be tuned by using cross-linking agents or by changing the physicochemical composition of the formulation.^45^ The mechanical properties for the 3D hydrogel scaffold are mainly evaluated as either elastic modulus (*E*) or shear modulus (*G*) by using rheology.^2^ A comprehensive review was done by Oyen *et al.*^49^ to discuss different mechanical characterisation techniques for hydrogels. Swelling property can also be used as an indication for hydrogel stiffness. The stiffer network shows lower swelling. Porosity is an important parameter for cell culture because the pore size can impact the perfusion of nutrients and oxygen in the hydrogel network. Lower swelling with higher modulus indicates the smaller pore size. An appropriate porosity of hydrogel scaffold is required to achieve the desired cell proliferation, migration, and differentiation.^46^ Smaller pore sizes can limit the cell migration towards the centre, distribution of nutrients, and removal of waste materials from the hydrogel network. However, larger pore size can reduce the total surface area for the cell attachment.^50^ The optimum pore size of the hydrogel scaffold is dependent on the specific tissue and cell. For example, scaffold with pore sizes between 200–400 μm was suitable for bone tissue formation, while 50–200 μm were required for smooth muscle cells.^51^ Hydrogel network with pore sizes between 10–75 μm can only form fibrous tissue, while pores greater than 100 μm enable vascularization. Pores larger than 400 μm reduce the total surface area, resulting in minimal cell–cell contact ratio.^52^ Topographical modification of the hydrogel surface can provide a better platform for cellular adhesion. Studies showed the direct influence of micro- and nano-topographical modification of the surface on cell adhesion and proliferation.^53–55^ A summary of different studies that evaluate the influence of mechanical properties of the hydrogels in 3D cell culture is provided in Table 1.

**Table 1:** Evaluation of the mechanical properties of the hydrogels for 3D cell culture

Hydrogels	Cell lines	Condition		Mechanical stiffness (*E*_m_: elastic modulus)	Pore size	Morphology of the cells	References
		Modifiers	Gel formation				
Collagen-I	Bone marrow-derived human mesenchymal stem cells	None	Rat tail derived type-I collagen was used to form hydrogels (pH ∼ 7.2) at different concentrations (1, 2, or 3 mg ml^−1^) by adjusting the volumes of collagen in 1 N NaOH, 10× PBS, and sterile distilled water	1 mg ml^−1^ gel: *E*_m_ = 113 ± 24.7 Pa	Mean pore areas were 0.317, 0.099, and 0.116 μm^2^ for 1, 2, and 3 mg ml^−1^ hydrogels respectively	-Spheroid organisation was observed	121
				2 mg ml^−1^ gel: *E*_m_ = 547.1 ± 79.1 Pa		-Significantly lower spreading and viability of the cells was observed on 3 mg ml^−1^ gel	
				3 mg ml^−1^ gel: *E*_m_ = 732.4 ± 50.6 Pa			
	Bone marrow-derived mesenchymal stem cells	Silk from *Bombyx mori*	The collagen pregel (pH-7.4 ± 0.4) was mixed with ice-cold pH-neutralized silk solution (70 mg ml^−1^) at different ratios of collagen and silk. In the hydrogel, the final collagen concentration was 7.8 mg ml^−1^. Four different types of composition were achieved: collagen : silk = 4 : 1, 2 : 1, 4 : 3, 1 : 1 that referred 25%, 50%, 75% and 100% of silk content	Pure collagen (7.8 mg ml^−1^): *E*_m_ = 0.62 ± 0.13 kPa	Not determined	More elongated cells compared to the pure collagen-I in all composites	122
				25% silk contain: *E*_m_ = 1.05 ± 0.15 kPa			
				50% silk contain: *E*_m_ = 1.26 ± 0.17 kPa			
				75% silk contain: *E*_m_ = 1.14 ± 0.03 kPa			
				100% silk contain: 1.31 ± 0.23 kPa			
	Bone marrow-derived human mesenchymal stem cells	Silk fibroin from *Bombyx mori*	-The collagen and silk fibroin (CS) ratio were fixed at 1 : 7 but the concentrations of both proteins changed	CSA: *E*_m_ = 0.017 kPa	Not determined	Cells showed polygonal morphology and no significant difference in the shape among the silk fibroin/collagen hydrogel groups was noticed	123
			CSA-0.35% silk fibroin + 0.5 mg ml^−1^ collagen	CSB: *E*_m_ = 0.279 kPa			
			CSB-0.5% silk fibroin + 0.71 mg ml^−1^ collagen	CSC: *E*_m_ = 0.385 kPa			
			CSC-0.7% silk fibroin + 1 mg ml^−1^ collagen	CSD: *E*_m_ = 1.15 kPa			
			CSD-1.05% silk fibroin + 1.5 mg ml^−1^ collagen	CSE: *E*_m_ = 1.19 kPa			
			CSE-1.4% silk fibroin + 2 mg ml^−1^ collagen	CSF: *E*_m_ = 1.53 kPa			
			CSF-1.75% silk fibroin + 2.5 mg ml^−1^ collagen	CSG: *E*_m_ = 5.16 kPa			
			CSG-2.1% silk fibroin + 3 mg ml^−1^ collagen	CSH: *E*_m_ = 6.81 kPa			
			CSH-2.45% silk fibroin + 3.5 mg ml^−1^ collagen				
			-Neutralisation of collagen-I at pH-7 and sonication of the silk fibroin was required for gel formation				
	Bone marrow-derived rat mesenchymal stem cells	Silica–tetramethyl orthosilane (TMOS)	The collagen solution (3.5 mg ml^−1^) was mixed with hydrolysed TMOS at weight ratios of collagen : TMOS = 90 : 10 (Col-10S) and 80 : 20 (Col-20S)	Col-10S: *E*_m_ ∼ 125 kPa	Not determined	Cells showed highly elongated morphology in both composites	124
				Col-20S: *E*_m_ ∼ 400 kPa			
	Bone marrow-derived human mesenchymal stem cells	Agarose	Collagen–agarose hydrogel blends were prepared with varying concentration of each polymer	AG0.5–COL0.21: *E*_m_ = 18.1 ± 3.5 kPa	Not determined	Highest spreading, elongation and osteogenic differentiation of MSC were observed in the softer gel	125
			AG0.5–COL0.21: 0.5 g ml^−1^ agarose + 0.21 g ml^−1^ collagen-I	AG1–COL0.10: *E*_m_ = 53.1 ± 10.3 kPa			
			AG1–COL0.10: 1 g ml^−1^ agarose + 0.10 g ml^−1^ collagen-I	AG2–COL0.05: *E*_m_ = 89.1 ± 13.9 kPa			
			AG2–COL0.05: 2 g ml^−1^ agarose + 0.05 g ml^−1^ collagen-I				
Gelatin	Chondrocytes	Methacrylic anhydride (MA)	Different volumes of MA (0.2 ml, 1 ml, and 5 ml) was added to 10% gelatin solution (pH-7.6)	0.2 ml MA: *E*_m_ = 3.8 ± 0.3 kPa	Not determined	-Rounded cells with no obvious F-actin stretch was observed in high stiff gel	126
				1 ml MA: *E*_m_ = 17.1 ± 2.4 kPa		-Elongated cells with abundant F-actin filaments was found in low stiff gel	
				5 ml MA: *E*_m_ = 29.9 ± 3.4 kPa			
	Sheep mesenchymal stem cells	Alginate	Gelatin (Gel)–alginate (Alg) gel was prepared by adding different concentration of alginate (1%, 3%, 5%, 7% and 9%) with the constant concentration of gelatin (6%)	6% Gel–5% Alg: *E*_m_ = 29.8 ± 2.49 kPa	Not determined	Not mentioned	127
				6% Gel–6% Alg: *E*_m_ = 34.4 ± 5.89 kPa			
				6% Gel–7% Alg: *E*_m_ = 39.6 ± 2.70 kPa			
	Preosteoblasts	-Methacrylic anhydride (MAA)	To prepare HA–GelMA hydrogel, different concentrations of HA (0, 1, 5, 20 mg ml^−1^) was added with 5% of GelMA	5% GelMA: *E*_m_ = 4.3 ± 0.2 kPa	Not determined	No significant changes in cell morphology was found in all composites, while higher proliferation was observed in composite with 20 mg ml^−1^ HA	128
		-Hydroxyapatite (HA)		1 mg ml^−1^ HA–5% GelMA: *E*_m_ = 4.8 ± 0.3 kPa			
				5 mg ml^−1^ HA–5% GelMA: *E*_m_ = 6.0 ± 0.8 kPa			
				20 mg ml^−1^ HA–5% GelMA: *E*_m_ = 9.3 ± 2.5 kPa			
Chitosan	Buffalo embryonic stem cell	Gelatin	-2% of chitosan solution was prepared by dissolving in 1% (v/v) acetic acid aqueous solvent	Pure chitosan: *E*_m_ = 5.5 ± 0.2 kPa	-Relatively spherical pores compare to the pure chitosan scaffold	Cells maintained polygonal morphology in all composites	91
			-2% gelatin solution was prepared by dissolving in deionized water	CG21: *E*_m_ = 47.9 ± 2.0 kPa	-Pore size of pure chitosan ranging from 50–100 μm		
			-The homogeneous mixture with different ratios of chitosan and gelatin was prepared to get the modified scaffold. Chitosan : gelatin = 2 : 1 (CG21), 1 : 1 (CG11), and 1 : 2 (CG12)	CG11: *E*_m_ = 49 ± 3.0 kPa	-Pore size of chitosan–gelatin scaffold ranging from 35–55 μm		
				CG12: *E*_m_ = 72.5 ± 5.0 kPa			
Alginate	Human adipose-derived stem cells	Chitosan	Alginate–chitosan hydrogel was prepared by adding different amount of chitosan in alginate solution (10%). The different mixing ratios of alginate : chitosan = 1 : 0, 1 : 0.2, 1 : 0.4, 1 : 0.6, 1 : 0.8, 1 : 0.9, 1 : 1.0, 1 : 1.1, and 1 : 1.2	-No significant difference in the elastic modulus among the hydrogels whose ratio of chitosan to alginate was 0.9–1.1 and the value was approximately 0.18 MPa	Not determined	Not mentioned	129
				-Elastic modulus decreased significantly in the ratio of 1 : 1.2 of chitosan to alginate and the approximate value was 0.12 MPa			
	Human breast cancer cells (MCF-7)	CaCl_2_	-0.5%, 1%, and 2% alginate gel was prepared by varying the CaCl_2_ content (0.2 M, 0.5 M or 1 M)	-A weaker cross-linker content (*i.e.* 0.2 M CaCl_2_) did not show significantly different stiffnesses among different concentrations of alginate	Not determined	-In softer gel (150–200 kPa), cells proliferated and formed steroids with a mean size of 100 μm	130
				-Gels with overlapping ranges of stiffness were merged as a unique range, to obtain four different categories of stiffness: soft gel-*E*_m_ ranging from 150–200 kPa, moderately stiff gel-*E*_m_ ranging from 300–350 kPa, medium stiff gel-*E*_m_ ranging from 900–1800 kPa, highly stiff gel-*E*_m_ ranging from 2500–4000 kPa		-In stiffer gel (>300 kPa), rounded cells with cluster organisation was noticed	
						-In moderately stiff gel (300–350 kPa), the cluster size was approximately (>10 μm)	
						-In medium stiff gel (900–1800), the cluster size was about 30 μm	
Fibrin	Human articular chondrocytes (hACs)	-Varying the concentration of fibrinogen	-Fibrin hydrogels were prepared by maintaining the different final concentrations of fibrinogen (15 mg ml^−1^, 27 mg ml^−1^, 50 mg ml^−1^) and constant concentration of thrombin (1 U ml^−1^)	15 mg ml^−1^: *E*_m_ = 1.1 ± 0.3 kPa	Not determined	Chondrocyte sphericity increased with higher elasticity. Cell morphology was more elongated in hydrogels with 1 kPa and 14 kPa elastic modulus compared to hydrogels with 32 kPa elastic modulus	131
				27 mg ml^−1^: *E*_m_ = 13.8 ± 1.3 kPa			
				50 mg ml^−1^: *E*_m_ = 31.8 ± 2.8 kPa			
Agarose	Nucleus pulposus cells	Bovine collagen-I	Different volume of 5 mg ml^−1^ collagen solution was added to 4% agarose solution to get the final concentration of 2% agarose and 4.5 mg ml^−1^ collagen or 2% agarose and 2 mg ml^−1^ collagen	4% agarose: *E*_m_ = 16.5 ± 0.2 kPa	Not determined	Rounded morphology was observed for both pure agarose and agarose–collagen composite	132
				2% agarose and 4.5 mg ml^−1^ collagen: *E*_m_ = 18.8 ± 0.4 kPa			
				2% agarose and 2 mg ml^−1^ collagen: *E*_m_ = 13.3 ± 0.6 kPa			
Polyacrylamide (PAA)	Bone marrow-derived mice mesenchymal stem cells	Bis-acrylamide	-Gel with different stiffness was prepared by adjusting the concentration of crosslinker bis-acrylamide (0.1%, 0.5%, and 0.7%) with acrylamide monomer (8%)	Gel containing 0.1% bis-acrylamide: *E*_m_ = 13–16 kPa (Soft gel)	Not determined	Soft gel: oval and short spindle shapes	133
				Gel containing 0.5% bis-acrylamide: *E*_m_ = 48–53 kPa (medium stiff gel)		Medium stiff gel: elongated shape	
				Gel containing 0.7% bis-acrylamide: *E*_m_ = 62–68 kPa (high stiff gel)		High stiff gel: polygonal shape	
Poly(ethylene)glycol (PEG)	Chondrocytes	Photo-initiator, 2-hydroxy-l-[4-(hydroxyethoxy)phenyl]-2-methyl-l-propanone	10%, 20%, and 30% (w/w) photopolymerised PEG hydrogel was prepared by adding 0.05% (w/w) of photo-initiator	10% gel: *E*_m_ = 34 ± 3 kPa	10% gel: 140 Å	Not mentioned	134
				20% gel: *E*_m_ = 360 ± 14 kPa	20% gel: 60 Å		
				30% gel: *E*_m_ = 940 ± 60 kPa	30% gel: 50 Å		
	Human articular chondrocytes (hACs)	Dextran	PEG–dextran hydrogels were generated using different ratios of PEG linker and dextran. PEG linker : dextran = 2.3 mM : 3 mM, 5 mM : 5.8 mM, and 7.5 mM : 8.2 mM	PEG linker : dextran	In gel with 7.5 mM PEG linker and 8.2 mM dextran, the pore size is less than 10 nm	Chondrocytes spherical morphology was observed in all elasticity	131
				2.3 mM : 3 mM, *E*_m_ = 1 ± 0.3 kPa			
				5 mM : 5.8 mM, *E*_m_ = 16.2 ± 1.8 kPa			
				7.5 mM : 8.2 mM, *E*_m_ = 29.6 ± 3 kPa			

## 5. Cell culture in hydrogel–microfluidic matrices

In *in vivo* condition, cells are exposed to the complex matrix-based microenvironment and adjoining cells that facilitates cellular communication and secretion.^56^ In traditional 2D cell culture techniques, cell growth has occurred on a flat dish and cells usually grow as a monolayer for adherent cells. Non-adherent cells are covered by the cellular media without any physically relevant microenvironment. Cellular morphology can change drastically because of the unconstrained cellular spreading and migration on a single plane.^57^ Changed morphology can affect the organisation of the organelles inside the cells.^3^ Moreover, the lack of cell-extracellular matrix interaction could initiate abnormal proliferation, polarisation, gene expression, protein secretion, and cell signalling.^3^ Several reports recognised that the transition from 2D cell culture to 3D hydrogel-matrix based cell culture leads to significant improvement in the cellular morphology, protein expression, differentiation, migration and functionalities in *in vitro* platforms.^58–61^ Unlike 2D culture, 3D cell culture in hydrogel scaffold provides more tissue like complexities and allows the spatial orientation of cells. Cells in 3D scaffold with low stiffness can grow in all directions and are able to maintain the physiological morphology.^62^ The 3D scaffold also provides integrin and growth factors' binding sites to maintain the cell–cell and cell–matrix interactions which regulate overall cellular function.^57^ An extensive review by Edmondson *et al.*^63^ discussed the characteristic comparison between 2D and 3D cell culture systems based on the cell growth condition, cell proliferation, gene and protein expression profiles. However, to mimic the highly complex dynamic *in vivo* microenvironment, the cell culture platform needs to provide a continuous supply of nutrition and oxygen along with the constant removal of waste as well as mechanical stimuli.^7^ The combination of microfluidic systems with 3D cell culture could address this bottleneck and introduce a new dimension in the study of cellular and tissue biology.^64–67^

**Fig. 5:**
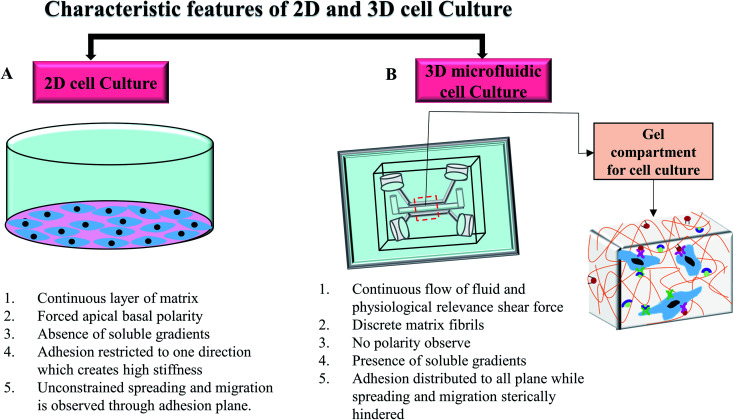
Characteristic attributes of 2D and 3D microfluidic cell culture systems. (A) Characteristics of the conventional 2D cell culture system. (B) A commonly used microfluidic 3D cell culture model and cellular orientation in the gel compartment.

Microfluidics system generally refers to the use of the small volume of fluids in microchannels or microchambers to create complex dynamic cellular microenvironments with tuneable mechanical properties such as shear, strain, and stress. Microfluidics allows for the reproducible simulation of perfusion flow, spatial control over co-cultures and signalling gradients, cell–cell and cell–matrix interaction in microchannel to mimic the physiological conditions in the 3D *in vitro* models.^68^ Jang *et al.*^68^ studied shear–stress induced osteoblast differentiation in 3D microfluidic chip. The study suggested a 10-fold increase of alkaline phosphatase (ALP) activity in MC3T3-E1 cells compared to the 2D cell culture. A microfluidic 3D chip-based drug sensitive platform was designed by using monoculture and co-culture of the SPCA-1 (human non-small cell lung cancer cell line) and the HFL1 (human lung fibroblast cell line) for the clinical individualised treatment of lung cancer.^69^ Both cell lines showed flat morphology with several protrusions on the cell surface in 2D culture while spherical tight junction was developed with several surrounded protrusions in chip. In the co-culture 3D model, HFL1 cells were able to capture the spherical SPCA-1 cells and developed clusters inside the chip. Another study grew human pancreatic ductal adenocarcinoma cells (PDACs) in a 3D microfluidic collagen-coated chamber and compared the morphology, growth characteristic, and chemotherapeutic responses of the cells to the 2D cell culture system.^70^ In this study, three PDAC cell lines including PANC1, BxPC3 and MiaPaCa2 were used. Cells exhibited slightly different morphology and adhesion phenomena for different cell lines in both 2D culture and 3D microfluidic chip. In 2D cell culture, BxPC3 cells showed stronger adhesion among three cell lines while all cells displayed epithelial morphology. Only BxPC3 formed uniform clusters of cells in 2D model. In collagen-coated chip, only BxPC3 exhibited collagen selective adhesion. BxPC3 and PDANC1 showed flat morphology with few spherical cells on top while MiaPaCa2 grew as spherical. All cell lines aggregated to form 3D clusters inside the chips. The efficacy of cisplatin on PANC1 cells were investigated in both 2D culture and 3D chip after 72 hours. The major limitation when testing chemotherapeutic medications in both preclinical and clinical models is the frequent lack of response when applying drug concentrations derived from standard 2D *in vitro* culture. In this study, the calculated IC_50_ at 3.25 ± 0.2 μM of cisplatin was achieved in 2D culture after 3 days. However, a higher cisplatin concentration was required to obtain the same reduced viability in chip. At the concentration of 100 μM cisplatin, the cell viability reduced by 30% in chip that resembled more *in vivo* like condition. This is consistent with data obtained from the *in vivo* study in which a high concentration of cisplatin was needed for cell growth inhibition. The complete growth inhibition of PANC1 cells was achieved with approximately 240 μM plasma concentration of cisplatin after 10 days.^71^ The results indicate that 3D cell culture in the chip resembled *in vivo* condition better. Fig. 5 shows the differences between cells grown on 2D and 3D microfluidic cell culture systems.

**Fig. 6:**
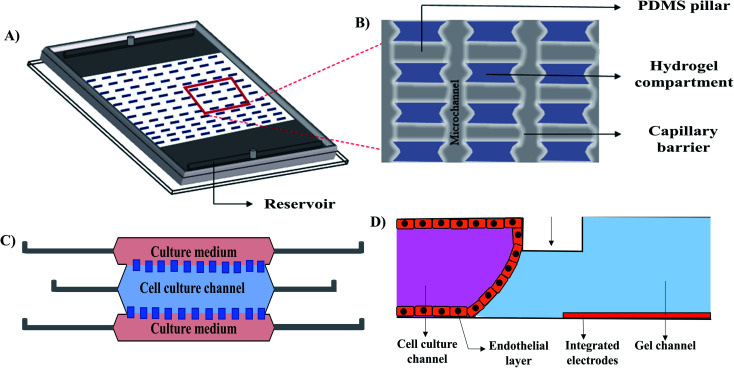
Reconstructed schematic diagrams of 3D microfluidic cell culture devices. (A) Isometric view of the hydrogel patterns with a zoomed in illustration (B) of the capillary barriers, PDMS pillars and hydrogel compartments of the compartmentalized model,^73^ (C) TEM (the tumour microenvironment) model,^74^ and (D) vascular model.^75^

Introducing hydrogels as an ECM into a microfluidic 3D cell culture system provides mechanical support because of its porosity, high water retention capability, and stiffness. ECM like hydrogels can promote cell proliferation, differentiation, migration, and protein secretion. The advantages of combining microfluidic cell culture systems with hydrogels are (i) fabricating microchannel at an appropriate dimension relevant to the *in vivo* environment, (ii) maintaining unprecedented temporal and spatial control over fluids and cellular distribution by creating reproducible biointerfaces of medium–matrix, and (iii) establishing control over signalling gradients for developing dynamic cellular microenvironment.^52,72^ Hydrogel based 3D microfluidic cell culture devices could be used to investigate cell proliferation, spreading, cell to cell contact, as well as *in vitro* drug screening. Gumuscu *et al.*^73^ extensively studied the compartmentalized hydrogel-based 3D microfluidic cell culture system using the Caco-2 cell line (Fig. 6A and B). A PDMS microchip with 500 individual collagen compartments, separated by microfluidic channels was used for observing long-term and parallel culture of Caco-2 human intestine cells with continuous fluid perfusion. The microchip was also used to observe cellular adhesion with intestinal pathogens and drugs that provided an *in vitro* platform for drug screening. A collagenase-based enzymatic method was developed to retrieve the embedded cells form the microfluidic TME (the tumour microenvironment) model (Fig. 6C).^74^ In this study, a transparent cyclic olefin polymer microfluidic device, composed of a central microchamber and two lateral microchannels was used. The central cell culture channel was coated with collagen hydrogel before cell infusion. HCT-116 colon carcinoma cell line and U251-MG glioblastoma cell line were independently infused through the channel. After 24 hours of incubation, cells were collected by enzymatic degradation. Following collagenase treatment, the cells were re-embedded and recovered after 72 hours for checking viability. Their technique allowed the repaid recovery of the cells within 10 min with high viability for both cell types. A 3D hydrogel based vascular model was designed by Wong *et al.*^75^ (Fig. 6D) to study the electrochemical permeability of endothelial cells in a microfluidic platform. The advantage of the design was that it allowed for the measurement of the endothelial permeability in the incubator environment without employing any complex optical instrument.

## 6. Commonly used hydrogels in microfluidic 3D cell culture

Hydrogels, used in microfluidic 3D cell culture system should ideally imitate the native tissue-specific microenvironment and deliberate cell attachment sites for the bioactivation of cellular factors that are responsible for cell proliferation, differentiation and migration. Alongside, the scaffold preparation procedure should not be harsh for maintaining cell viability and functionality.^76^ This section discusses natural and synthetic hydrogels commonly used in microfluidic cell culture systems. Table 2 provides a summary of different hydrogels with their properties, limitations, and applications in microfluidic 3D cell culture systems.

**Table 2:** Widely used hydrogels as ECM in the microfluidic 3D cell culture system

Hydrogel	Origin	Type	Chemical composition	Gelation	Remarkable features	Limitations	Cell lines	Reference
Collagen-I	Protein-extracellular matrix	Natural	A triple helical structure composed of two identical polypeptide chains (α1) and slightly different additional polypeptide chain (α2)	Thermo-responsive	-Biocompatible	-Long-term stability issues	Human adult dermal microvascular endothelial cells	135
					-Biodegradable		Fibroblast	22 and 136–138
					-Porosity	-Batch-to-batch variability	Neural stem cells (C17.2)	117
					-Collagen to cell ligand binding		HUVECs	79 and 82
							Neuron cells	80
							Human colon carcinoma cell line	74 and 83
							Human adipose stem cells	139
							Human induced pluripotent stem cells	81
							Glioblastoma cell line	74
Gelatine	Protein-extracellular matrix	Natural	Derived from the thermal denaturalization of collagen and consists of a large number of glycine, proline, and 4-hydroxy proline residues	Thermo-responsive	-Biocompatible	-Poor mechanical properties	Porcine aortic valvular interstitial cells	86 and 87
					-Biodegradable	-Susceptible for enzymatic degradation	Cardiomyocytes	88
					-Allow structural modification by reacting with different biomaterials	-Poor solubility in higher concentration	Osteoblast	140
							Chondrocytes	141
							Fibroblast	142
Chitosan	Polysaccharide-crustaceans	Natural	β-(1→4)-Linked-d-glucosamine and *N*-acetyl-d-glucosamine	pH-responsive	-Biodegradable	Poor solubility	Cardiomyocytes	21
					-Biocompatible			
					-Non-toxic		HUVECs	92
					-Chelating agent			
Fibrin	Protein-blood	Natural	Consisted of three pairs of polypeptide chains, designated Aα, Bβ and γ	Enzymatic	-Biocompatible	Easily degraded by proteases	Colorectal cancer and gastric cancer cells	94
					-Biodegradable			
					-Easy tunability			
Agarose	Polysaccharide-seaweed	Natural	1,4-Linked 3,6-anhydro-l-galactose and 1,3-linked-d-galactose derivatives	Thermo-responsive	-High gel strength at low concentration	-Non-biodegradable	Oral cancer cells	98
					-Gelling and melting temperature can be modified by chemical modification	-Requires adhesive ligands to enable cell attachment	Chondrocyte	99 and 100
					-Biocompatible		Human colorectal adenocarcinoma	101
							HepG2 cells	103
							Hela cells	104
							Human oral cancer cells	102
							Colon cancer cells	105
Alginate	Polysaccharide-seaweed	Natural	Linear copolymers containing blocks of 1,4-linked β-d-mannuronate (M) and α-l-guluronate (G) residues	Ionotropic	-User flexibility to alter molecular weight, composition, and macromolecular composition	-Requires adhesive ligands to enable cell attachment	Human breast cancer cells	108
					-Biocompatible	-Non-biodegradable		
					-Low toxicity			
Hyaluronic acid (HA)	Polysaccharide-extracellular matrix	Natural	β-1,4-d-Glucuronic acid and β-1,3-*N*-acetyl-d-glucosamine	-Thermo-responsive	-Able to modify hyaluronic acid with many functional groups	-Large size constructs	Fibroblast	110
				-Photo responsive	-Biocompatible	-Nonadhesive		
					-Nonimmunogenic			
					-Enzymatic degradation			
Poly(ethylene glycol) (PEG)		Synthetic			-Hydrophilic	Lack of endogenous factors that promote cell behavior	Mesenchymal stem cells	112
					-Inert			
					-Can be cross-linked with many functional groups			
Polyacrylamide (PAA)		Synthetic			Ability to fine tune stiffness	-Require toxic cross-linkers	Bacterial and yeast cells	113
						-Lengthy gel preparation technique		
						-Suitable for only small batches		
						-Suitable to use in 2D cell culture		

### 6.1. Collagen

Collagen, especially type I, is the most commonly used hydrogel in cell culture.^77^ Generally, self-assembly of collagen fibrils occurs rapidly at higher temperatures and neutral pH. Properties such as biocompatibility, biodegradability, porosity, and collagen-to-cell ligand binding make collagen an excellent choice to study cell encapsulation and cell migration. Collagen microstructure has the direct influence in the tissue specific mechanical parameters and in the cellular migration. It is important to evaluate some microscopic key properties, such as stiffness and porosity before architecting tissue specific collagen scaffold for 3D cell culture.^78^ One of the benefits of using collagen for microfluidic cell culture is the ability to establish chemical and biological gradients. Collagen scaffold is used as robust platforms for culturing different cell lines to construct *in vivo* relevant tumor microenvironments,^22,79^*in vitro* neural networks,^80^*in vitro* microvascular networks,^81,82^ and *in vitro* human intestinal villi^83^ in microfluidic devices. Shim *et al.*^83^ aimed to build a human intestinal villi shaped collagen scaffold on a chip and to introduce fluid shear alongside to check the influence of gut microenvironment on the physiological functions of the human colon carcinoma cells (Caco-2) line. The study compared the cellular morphology and the vital functions of Caco-2 cells in 3D microfluidic gut chip, 2D monolayer culture on transwell, and 2D monolayer culture on microfluidic chips. Cells showed prismatic morphology in the 3D microfluidic gut chip, while the cells were flattened and attached closely to the membrane in the 2D well and 2D chip. In addition, Caco-2 cells showed enhance metabolic functions on 3D microfluidic gut chip. Caco-2 cells showed 7-fold increase in P450 3A4 enzymatic activity in 3D microfluidic gut chip than the 2D well and 2D chip.

### 6.2. Gelatin

Gelatin is derived from the hydrolysis of collagen. It is abundantly used in the biomedical research because of its structural similarities to ECM, availability, cost effectiveness, excellent biodegradability and biocompatibility. Gelatin is the commonly used biopolymer in 3D cell cultures because of its ability to host a variety of cell lines by proving suitable chemical and biological binding motifs.^84^ Additionally, different functional groups, present in the chemical structure of gelatin, allows the chemical modification by reacting with other biomaterials. Gelatin-based 3D microfluidic platforms are fabricated to emulate the cellular microenvironment for long-term cell culture and growth.^85^ Chen *et al.*^86^ utilized gelatin-methacrylate to study cell-to-cell interactions between pancreatic and valvular endothelial and interstitial cells. Gelatin (10%) and methacrylic anhydride (94%) were mixed to achieve the highest degree of methacrylation, and porcine aortic valvular intestinal cells were mixed in a 1 : 1 ratio with the gelatin methacrylate hydrogel. The effects on embedded cell viability were determined by varying gelatin methacrylate concentrations, photo-initiator concentrations, and ultra-violet curing time. The encapsulated cells in the hydrogel were then injected into the microfluidic device. The vascular endothelial cells suppressed vascular interstitial cells in myofibroblast differentiation, and this effect was enhanced by the shear stress.^86^ These results were also consistent with the macroscale tissue-engineered model of Butcher *et al.*,^87^ thus confirming the *in vitro* fidelity of the microfluidic culture system. In another study, gelatin and tropoelastin-based hydrogel were used to coat the surfaces of microchannels to generate a hydrogel layer on the channel walls.^88^ Rat cardiomyocytes seeded on these hydrogel layers showed preferential attachment and higher spontaneous beating rates on tropoelastin coatings as compared to gelatin. Cardiomyocytes respond in favour of elastic, soft tropoelastin culture substrates. This study showed the potential use of tropoelastin-based hydrogels as a suitable coating for some organ-on-a-chip applications and limited the use of gelatin for certain cell lines. However, some drawbacks including: poor stiffness without crosslinkers, susceptibility to enzymatic degradation and poor solubility with higher viscosity in concentrated solution, restrict the application of gelatin to a greater extent.^84^

### 6.3. Chitosan

Chitosan is the derivative of chitin polysaccharide. Chitosan usually requires modification to improve its physicochemical properties. Chitosan bears a close structural resemblance to mammalian polysaccharides that makes it an attractive choice for cell encapsulation.^89^ However, before chitosan hydrogels can be used, its properties such as stability and durability need to be improved by polymeric cross-linking. Furthermore, chitosan stiffness strongly affects the cell morphology. Elongated cells were observed in gel with stiffness greater than 5 kPa while abnormal circular morphology was seen in the less stiff gel (<5 kPa).^90,91^ Chitosan hydrogel has been used as a scaffold to mimic the *in vitro* cardiac tissue microenvironment.^21^ Chitosan (1%) was used to culture cardiomyocytes on microfluidic chip. Cell medium perfusion was carried out at the rate of 0.1 ml h^−1^ after the attachment of the cells to the hydrogel scaffold. Cells grown on the chip in microscale had higher confluency compared to those in two-dimensional culture. From the tissue construct staining analysis, an integrated organoid-like tissue was also shown after ten days of culture. A tunable microstructured membrane-on-chip was made by blending poly(ε-caprolactone)–chitosan (PCL–CHT) in different ratios to analyse the effect of mechanical parameters of the ECM structure in cell growth and proliferation.^92^ In this study, HUVECs were seeded on the channel coated with the modified gel for 3 days before analysing. The results suggested that the membrane with a large surface pore facilitated the cell incorporation while interconnected small porous structure was suitable for nutrients and oxygen diffusion, along with the waste removal. Furthermore, the study showed an improved resistance of cellular barrier with the higher percentage of chitosan.

### 6.4. Fibrin

Fibrin is a natural biopolymer formed by the polymerization of fibrinogen, which is isolated from blood plasma. First, fibrinogen is converted into fibrin monomer in the presence of thrombin and CaCl_2_. Then fibrin monomers are self-assembled by hydrogen bonds to form insoluble fibrin. Additional cross-linking is provided by the blood coagulation factor XIIIa to produce fibrin mesh. Properties such as biocompatibility, haemostasis, and biodegradation make fibrin as an excellent choice for studying *in vitro* wound healing. Moreover, fibrin gel morphology, gelation time, and stability could be easily tuned by changing the concentration of thrombin and the blood coagulation factor XIIIa.^93^ Ahn *et al.*^94^ introduced a microfluidic bone mimicking microenvironment using hydroxyapatite–fibrin hydrogel composite to recapitulate a 3D vascularized tumour spheroids condition. Hydroxyapatite is the essential component of the bone and teeth which gives them the rigidity. The composite was made by adding hydroxyapatite in different concentrations (0.0, 0.2, and 0.4%) with fibrin hydrogel to study the mechanical properties of ECM on the bone microenvironment. The authors seeded colorectal cancer (SW620) and gastric cancer (MKN74) cells in the developed composite material. The team successfully investigated the viability, morphology, proliferation, and migration of the cells as well as studied the cell–cell and cell–matrix interaction. The cell viability, morphology, and proliferation of both cell types depend on rigidity *i.e.* the concentration of hydroxyapatite. With the higher concentration of hydroxyapatite, both cell lines showed significantly reduced migration with less blood vessel sprouts during angiogenesis.

### 6.5. Agarose

Agarose is a marine-based linear polysaccharide. It is extracted from seaweed and composed of alternating copolymers of (1–3)-linked β-d-galactose and (1–4)-linked (3–6)-anhydro-α-l-galactose.^95^ This thermo reversible polymer can induce gelation or liquify at different temperatures varying from 20 °C to 70 °C, depending on the molecular weight and the degree of hydroxyethylation.^96^ Due to the temperature-sensitive water solubility of this polymer, it is suitable for the cell encapsulation. Agarose does not provide active anchor for cells that limit its use in the adherent cell culture.^97^ Microfluidic agarose-based cell encapsulation has been introduced as a promising platform for developing perfusion based microbioreactors for stable and high throughput cell culture,^98–100^ cell culture-based chemosensitivity assay,^101,102^ novel quantum dot cytotoxicity assay,^103^ and electric cell-substrate impedance sensing (ECIS) technique for real-time cell viability assay and drug screening.^104^ A leaf inspired agarose based 3D cell culture system was developed by Fan *et al.*^105^ for designing artificial microvascular network. The main purpose was to build up a branching perfusion model by using porous agarose hydrogel for the even distribution of oxygen and nutrients that support long term cells growth. HCT116 cells encapsulated agarose gel was used to construct multi-layered 3D vascular network. The encapsulated cells were able to absorb nutrients from the embedded vascular network and continued to grow for at least 2 weeks.

### 6.6. Alginate

Alginate is a natural polysaccharide derived from brown algae that forms a reversible gel in the presence of divalent cations *via* ionic crosslinking. Alginate hydrogel is notable for the flexibility to alter molecular weight, composition, macromolecular composition,^106^ and customized stiffness and pore sizes.^107^ An electrowetting on dielectric (EWOD) digital microfluidics (DMF) 3D cell culture system was developed by George *et al.*^108^ The aim of their design was to merge the EWOD DMF's multiplexing principle for uniform cell seeding in alginate hydrogels and using the device as a chemical screening platform. Although the main hurdles of the alginate on chip are the lack of microchannel compatible cross-linking methods of gelling and maintaining the cell viability throughout the whole process. The most common method for gelation of alginate is to introduce an acidifier to the alginate solution for lowering the pH of the system. pH of the system is dropped by releasing divalent calcium ions (Ca^2+^) from the pH sensitive alginate chelates. Cell encapsulation technique that relies on the sudden reduction of pH shows poor cell viability, even though, cells expose to the low pH only for few minutes.

### 6.7. Hyaluronic acid (HA)

Hyaluronic acid (HA) is a non-sulphated glycosaminoglycan composed of a repeating disaccharide unit of glucuronate and *N*-acetylglucosamine. Hyaluronic acid has several significant advantages as a hydrogel platform, including its biological relevance and chemical tunability. HA is distributed throughout many tissues, including skin, cartilage, and brain, and is known to play an important role in development, wound healing, and disease.^109^ A microfluidic device was fabricated by photocrosslinkable HA for studying cell immobilization.^110^ NIH-3T3 cell line was used in that study. They applied two approach for analysing cell viability in their developed microstructure. First, they used their fabricated microstructure as a docking template for the cellular attachment. Second, they directly encapsulated the cells into the microstructure before device fabrication. In the first approach, >99% of cells were viable after 24 hours and the cells remained viable up to 7 days. In the second approach, 82 ± 5% viability was observed 6 hours after encapsulation. For both approaches, cells could be recovered for further use.

### 6.8. Poly(ethylene glycol)

Poly(ethylene glycol) (PEG) is hydrophilic and a relative inert polymer. Like other hydrogels, PEG can be cross-linked with different functional groups, allowing user flexibility. Due to this flexibility, PEG has been used in many cell culture applications such as stem cell differentiation and angiogenesis.^111^ Liu *et al.*^112^ utilized a controlled cellular microenvironment in a microfluidic device for precise spatial diffusion of biomolecules. In their study, they used a gradient generating PDMS microfluidic device to immobilize the gradient of a cellular adhesive Arg–Gly–Asp peptide (RGD) on PEG hydrogels. A fluid dynamic model was simulated for the distribution of biomolecules and gradient generation in the device. PEG hydrogel was covalently bind with the RGD and then bone marrow-derived rat mesenchymal stem cells (MSCs) were cultured on the surface of the immobilized RGD gradient PEG hydrogel. Their result suggested a proportional increase of cell adhesion and distribution on treated PDMS surface with RGD concentration. They also determined the critical RGD concentration ranging from 0.107–0.143 mM for maximum MSCs adhesion on PEG hydrogel.

### 6.9. Polyacrylamide

Nghe *et al.*^113^ fabricated a microfluidic device with polyacrylamide (PAA) hydrogel as a biomaterial for bacterial and yeast cells growth. They compared different device designs with PAA to get the consistent spatio-temporal microenvironment for the cells. Their device showed consistency of getting a monolayer of cells which allowed easy microscopic observation. According to their study, the characteristic features of using PAA were that the gel was stable during fabrication and did not break like agarose gel, and PAA device did not need any chemical modification for improving cell adhesion like PDMS device. However, PAA hydrogels are formed by reacting acrylamide monomer with bisacrylamide crosslinker in the presence of ammonium persulfate (APS) and tetramethylethylenediamine (TEMED).^114^ These chemical crosslinkers generate free radicals, which cause cell toxicity. For this reason, PAA is not suitable for cell encapsulation. However, the advantage of PAA is its ability to fine-tune stiffness of the hydrogel. Thus, PAA is usually reserved for 2D studies where the stiffness of the hydrogels affects cell motility, spreading, and differentiation.

### 6.10. Combination of different hydrogels

The blending of different natural and synthetic hydrogels offers the capability of tuning mechanical and hydrogelation kinetics, along with the flexible incorporation of adhesive moieties for facilitating optimal culture conditions.^76,115^ Furthermore, hydrogel polymers can be tuned to mould desirable stiffness and internal structures.^116^ Collagen/fibrin hydrogels were used by Lee *et al.*^117^ to construct an artificial tissue for delivering growth factors and promoting migration and proliferation of murine neural stem cells. The mixture of HA/collagen improved the adhesion, migration, and proliferation of human umbilical vein endothelial cells in microfluidic platform.^118^ Agarose/collagen hydrogel composition was used to engineer a perfusable microfluidic network by using a bottom-up approach for sequential endothelial cell seedings.^20^ In that study, the spatially interconnected microchannels were used to build a fluid pathway to deliberate culture medium for the cellular distribution throughout the hydrogel scaffold. A hydrogel-based interpenetrating polymer network (IPN) was used to develop an endothelialised microvascular-on-chip by Qiu *et al.*^116^ In this study, agarose/gelatin composite was used to maintain the surrounding environment of endothelial cells (ECs) by imitating vascular stiffness and basement membrane functionality. The composite was suitable for successful EC seeding and ECs were appropriately assembled on self-deposing basement membrane protein (laminin- and collagen IV). The confluency of the cells was achieved within two days and maintained a semi-permeable barrier in the scaffold for more than a month under continuous laminar flow conditions. A multi-organoid body-on-a-chip model was constructed by Rajan *et al.*^119^ for analysing drug efficacy and toxicity. HA/gelatin-based hydrogel composite was used as extracellular matrix for cell seedings to build up miniaturized structure of the organ. Initially, a three organoid device was designed by embodying of liver (cell types: primary human hepatocytes, hepatic stellate cells and Kupffer cells, human liver-derived endothelial cells), cardiac (cell types: iPSc-derived cardiomyocytes, human cardiac fibroblast and cardiac endothelium cells) and lung (cell types: alveolar epithelial cells, normal human lung fibroblast, primary normal human bronchial epithelial cells) constructs. Later on, another three humanized constructs were integrated including blood vessel (cell types: human umbilical vein endothelial cells), testis (cell types: spermatogonial stem cells, Leydig cells, Sertoli cells, and peritubular cells), and brain (cell types: primary human brain microvascular endothelial cells, human brain vascular pericytes, human astrocytes, human microglial, human oligodendrocytes, and human neural cells) to expand the study. The design was able to maintain the highest viability of all types by using the common circulation for 21 days in three-organoids system, while the highest viability was observed for 14 days in the expanded six-organoids device.

## 7. Current challenges and future perspective

Hydrogels as an extracellular matrix scaffold show a promising advantage in microfluidic 3D cell culture. However, it is still challenging to achieve a standardized fabrication strategy that can maintain the batch to batch consistency for stable and highly functionalized tissue-specific hydrogels. Some challenges of hydrogel-based cell culture could overcome by incorporating microfluidic systems such as spatial distribution and regulation of cells, gradients, growth factors, and ligand. However, this approach also faces difficulties of getting a properly validated working protocols. Common problems associated with 3D hydrogel-based microfluidic systems are difficulties in hydrogel infusion through microchannels, formation of similar gel microstructures inside the channel, and reproducible medium-gel interference. Regulation of fluid through microchannels cannot fully mimic the microvascular complexity of native tissues and has issues for mimicking the spatial orientation of cells in layered tissue motif. A contradiction has also been noted by researchers as to whether or not microfluidic systems can create long-term clinically relevant data because of the use of the small number of cells in microchannel while reproducibility and device validation always are in question.^120^ Moreover, some technical issues are limiting the popularity of this novel approach among researchers, such as inadequate resources and facilities for chip fabrication and complexity in handling.

Facile fabrication and handling techniques with the integration of complicated *in vitro* tissue like conditions should be blended to overcome the challenges and utilize the benefits of microfluidic cell culture systems. Diverse tunable anisotropic hydrogels could provide native tissue-like complexity and an organ-like microenvironment for the heterogeneities of the cell behaviour.^117^ This condition might be achieved by combining the study of bio-orthogonal chemistry and organoid techniques. The organoid technique is the *in vitro* development of the miniature or simplified version of the organ. Bio-orthogonal chemistry refers to any particular reaction occurs in the living system without interfering native biochemical process. Applying these methods in a microfluidic device might introduce chemoselective groups on the cell surface, which eventually reacted with the chemoselective groups of other cell types and formed an organ-like tissue complexity on the chip. A combination of stimuli-responsive polymers with easily tunable synthetic polymers might provide the new way of removing the static stiffness and inducing the dynamic stress/strain surface interaction with cells to provide physiological tissue-like consistency. In future, micro-sensing devices could be integrated inside the microdevice for direct monitoring and analysis. Integrating pumping devices on chip might also reduce the analysis and validation error.

## 8. Conclusion

Hydrogels are promising in *in vitro* 3D scaffolds for cell culture because of their ability of recapitulate the extracellular microenvironment and providing mechanical support for cell adhesion, spreading, and proliferation. Undoubtedly 3D hydrogel-based microfluidic cell culture systems are a revolutionary shift from 2D cell culture for depth understanding in cellular biology and tissue engineering. The success of cell culture in microchannels mostly depends on the selection and fabrication of a preferred hydrogel as ECM structure. The hydrogel matrix composition can be manipulated to control the biochemical and biophysical properties of the hydrogel so that it is suitable for a specific cell line. Natural polymer-based hydrogels have different endogenous factors that facilitate a more organ-like microenvironment for cellular activities while synthetic hydrogels provide easy tunability and batch to batch consistency. Furthermore, natural hydrogels are biodegradable, biocompatible, and non-toxic, whereas synthetic hydrogels, in many cases, can be cytotoxic. Recently, smart hydrogels or stimuli-responsive hydrogels have aroused considerable interest because of their dynamic transition in response to external stimuli. To optimize the organ-like complexity with minimal cytotoxicity, it is crucial to synthesise hydrogels in different combination ratios of natural and synthetic polymers. The incorporation of stimulus-responsive components in the polymeric network can also facilitate the construction of more tissue-specific matrix. With lots of challenges, the future of the 3D microfluidic cell culture technique lies in the selection and the combination of high throughput technologies along with the easy and cost-effective fabrication and handling techniques. A proper validated, reproducible protocol can make this technique a great source of producing clinically relevant data to aid in our understanding of diseases processes and in the discovery and development of new therapeutic agents.

## List of abbreviations

2D: Two dimensional
3D: Three dimensional
APS: Ammonium persulfate
CLEX: Competitive ligand exchange crosslinking
ECM: Extra cellular matrix
ECIS: Electric cell-substrate impedance sensing
HA: Hyaluronic acid
IPA: Interpenetrating polymer network
LCST: Low critical temperature
MSCs: Mesenchymal stem cells
PAA: Polyacrylamide
PDMS: Polydimethylsiloxane
PEG: Poly(ethylene glycol)
PVA: Poly(vinyl alcohol)
TEM: The tumor microenvironment
TEMED: Tetramethylethylenediamine
UCST: Upper critical solution temperature

## Conflicts of interest

Nothing to declare.

## Supplementary Material
